# A Genetic System for *Methanocaldococcus jannaschii*: An Evolutionary Deeply Rooted Hyperthermophilic Methanarchaeon

**DOI:** 10.3389/fmicb.2019.01256

**Published:** 2019-07-03

**Authors:** Dwi Susanti, Mary C. Frazier, Biswarup Mukhopadhyay

**Affiliations:** ^1^Department of Biochemistry, Virginia Tech, Blacksburg, VA, United States; ^2^Biocomplexity Institute, Virginia Tech, Blacksburg, VA, United States; ^3^Virginia Tech Carilion School of Medicine, Virginia Tech, Blacksburg, VA, United States

**Keywords:** genetics, ancient archaea, methanogen, hyperthermophile, anaerobe, *Methanocaldococcus jannaschii*, gene knockout, protein overexpression

## Abstract

Phylogenetically deeply rooted methanogens belonging to the genus of *Methanocaldococcus* living in deep-sea hydrothermal vents derive energy exclusively from hydrogenotrophic methanogenesis, one of the oldest respiratory metabolisms on Earth. These hyperthermophilic, autotrophic archaea synthesize their biomolecules from inorganic substrates and perform high temperature biocatalysis producing methane, a valuable fuel and potent greenhouse gas. The information processing and stress response systems of archaea are highly homologous to those of the eukaryotes. For this broad relevance, *Methanocaldococcus jannaschii*, the first hyperthermophilic chemolithotrophic organism that was isolated from a deep-sea hydrothermal vent, was also the first archaeon and third organism for which the whole genome sequence was determined. The research that followed uncovered numerous novel information in multiple fields, including those described above. *M. jannaschii* was found to carry ancient redox control systems, precursors of dissimilatory sulfate reduction enzymes, and a eukaryotic-like protein translocation system. It provided a platform for structural genomics and tools for incorporating unnatural amino acids into proteins. However, the assignments of *in vivo* relevance to these findings or interrogations of unknown aspects of *M. jannaschii* through genetic manipulations remained out of reach, as the organism was genetically intractable. This report presents tools and methods that remove this block. It is now possible to knockout or modify a gene in *M. jannaschii* and genetically fuse a gene with an affinity tag sequence, thereby allowing facile isolation of a protein with *M. jannaschii*-specific attributes. These tools have helped to genetically validate the role of a novel coenzyme F_420_-dependent sulfite reductase in conferring resistance to sulfite in *M. jannaschii* and to demonstrate that the organism possesses a deazaflavin-dependent system for neutralizing oxygen.

## Introduction

*Methanocaldococcus jannaschii* is the first known hyperthermophilic methanogen (Jones et al., 1983). This phylogenetically deeply rooted archaeon is also the first hyperthermophilic chemolithotrophic organism that was isolated from a deep-sea hydrothermal vent (Jones et al., 1983) where the environmental conditions mimic those of early Earth (Jones et al., 1983; Jannasch and Mottl, 1985). *M. jannaschii* derives energy solely from hydrogenotrophic methanogenesis (4H_2_ + CO_2_ → CH_4_ + 2H_2_O), which is one of the most ancient respiratory metabolisms on Earth and developed most likely 3.49 billion years ago (Jones et al., 1983; Jannasch and Mottl, 1985; Ueno et al., 2006). It also generates the entire cell from inorganic nutrients (Jones et al., 1983) and, therefore, represents a minimal requirement for life to exist independent of other living systems.

Due to the special features mentioned above, *M. jannaschii* was the first archaeon and third organism for which the whole genome sequence was determined (Bult et al., 1996). Analysis of the sequence data revealed many novel metabolic features as well as genomic basis for known special features of the archaea (Bult et al., 1996). However, for 60% of the genes, even a predicted function could not be assigned (Bult et al., 1996). These findings and the option of making genome-based inquiries catalyzed vigorous research with *M. jannaschii* that yielded major breakthroughs.

Work with purified cellular parts and recombinant forms of proteins of *M. jannaschii* provided robust validation of previous observation that archaeal DNA replication, transcription, translation, and stress management machineries are simpler forms of respective eukaryotic systems (Olsen and Woese, 1996; Koonin et al., 1997; Reich et al., 2001; Grohmann and Werner, 2011). Similar parallels were found for eukaryotic protein translocation systems (Van den et al., 2004; Suloway et al., 2012). A structural genomics program leveraged *M. jannaschii* proteins for the discovery of new protein folds and molecular functions of newly identified proteins (Kim et al., 2003; Shin et al., 2007). The organism provided a platform for the elucidation of the pathways for the biosynthesis of coenzymes that are specific or key to methanogenesis (Graham and White, 2002). A novel tRNA-based cysteine biosynthesis system was discovered in an effort to understand how *M. jannaschii* generates this amino acid (Sauerwald et al., 2005), and specific tRNA-aminoacyl-tRNA synthetase pairs from *M. jannaschii* assisted the effort to introduce unnatural amino acids and imparting new functions to proteins (Wang and Schultz, 2001).

Genome-guided physiological studies led to novel hydrogen and redox-controlled systems of ecological and evolutionary biology relevance in *M. jannaschii* (Mukhopadhyay et al., 2000; Johnson and Mukhopadhyay, 2005; Susanti and Mukhopadhyay, 2012; Susanti et al., 2014, 2016). In particular, the results of these studies led to the hypotheses that methanogenesis and dissimilatory sulfate reduction system, another ancient respiratory metabolism on Earth (Shen et al., 2001), have intertwined evolutionary histories (Susanti and Mukhopadhyay, 2012), and the mechanism of redox regulation of metabolism in methanogens that is key to the function of numerous gut microbial systems has roots in the hydrothermal vent life of ancient organisms such as *M. jannaschii* (Mukhopadhyay et al., 2000; Johnson and Mukhopadhyay, 2005; Susanti and Mukhopadhyay, 2012; Susanti et al., 2014, 2016).

Most of the above-mentioned post-genome studies have utilized recombinant proteins generated in *Escherichia coli* and few utilized cellular parts of *M. jannaschii* or direct physiological measurements in *M. jannaschii* cultures. The latter efforts were facilitated by a method for a bioreactor-based cultivation of the organism under controlled conditions, which was developed soon after the sequencing of *M. jannaschii* genome (Mukhopadhyay et al., 1999). Several valuable inferences on the *in vivo* relevance of these discoveries have been made using a surrogate system, *Methanococcus maripaludis*, a mesophilic marine organism that is closely related to *M. jannaschii* and genetically tractable (Tumbula et al., 1997; Moore and Leigh, 2005). Similar benefit has also come from work with *Methanosarcina* species, which are amenable to sophisticated genetic analysis (Metcalf et al., 1997; Nayak and Metcalf, 2017). However, an interrogation of *M. jannaschii* genes with unknown or tentatively assigned functions in the organism itself remained unattainable, as this archaeon has been intractable genetically. In this communication, we report that we have filled this gap by developing tools and methods for genetic analysis of *M. jannaschii*, which provide two important genome manipulation capabilities: to construct in-frame gene deletion and enable homologous overexpression of a protein with an affinity tag. The former will allow finer analysis of gene functions. The latter would allow facile purification of *M. jannaschii* proteins produced in the most physiologically relevant location, in the organism itself, for *in vitro* studies.

## Materials And Methods

### Materials, Organisms, and Reagents

*M. jannaschii* JAL-1 (Jones et al., 1983) was from the laboratory stock (Johnson and Mukhopadhyay, 2005), which was derived from a culture that was a gift of Prof. Ralph S. Wolfe from the University of Illinois at Urbana-Champaign (Urbana, IL). *M. jannaschii* DSM 2661 was obtained from Leibniz-Institut DSMZ-Deutsche Sammlung von Mikroorganismen und Zellkulturen GmbH or DSMZ (Braunschweig, Germany). *Methanocaldococcus* strain FS.406-22 (MFS.406-22) (Mehta and Baross, 2006) was a gift from Prof. Robert Blankenship of the Washington University (St Louis, MO). *Escherichia coli* Stellar™ was obtained from Takara Bio (Mountain View, CA) and DNA oligonucleotides and synthetic DNA were from Integrated DNA Technologies, Inc. (Coralville, IA). The antibiotics and base analogs were obtained from the following sources: puromycin, neomycin, simvastatin, novobiocin, 6-methylpurine, 6-thioguanine, 6-azauracil, and 5-fluorouracil (MilliporeSigma, Burlington, MA); 8-azahypoxanthine (TCI America, Portland, OR); 8-aza-2,6-diaminopurine (Santa Cruz Biotechnology, Inc., Dallas, TX); and mevinolin (LKT laboratories, Inc., St. Paul, MN). Coenzyme F_420_ was purified from *Methanothermobacter thermautotrophicus* and F_420_H_2_ was generated by reducing F_420_ with sodium borohydride as described previously (Purwantini and Daniels, 1996; Susanti et al., 2016). All other reagents and chemicals were purchased from standard suppliers.

### Media and Growth Conditions

*M. jannaschii* strains were grown in medium 1 with a H_2_ and CO_2_ mixture (80:20, v/v) as methanogenesis substrates at 80°C as described previously (Mukhopadhyay et al., 1999). In brief, for growth in liquid medium, a 160 or 530 ml serum bottle (Wheaton Science Products, Millville, NJ) containing 10 or 200 ml anaerobic and sterile medium, respectively, sealed with a butyl rubber stopper and an aluminum crimp and pressurized with a mixture of H_2_ and CO_2_ (80:20, v/v) to 3 × 10^5^ Pa, was used. The inoculated culture was incubated in a shaker incubator (Lab Line Orbit Environ Shaker 3527, Melrose Park, IL) at 80°C and 200 rpm. Solid medium plates were prepared as follows. First, a sealed 160 ml serum bottle containing 50 ml of medium 3 (medium 1 lacking MgCl_2_.6H_2_O and CaCl_2_.2H_2_O) and Gelrite^®^ (Sigma Aldrich, St. Louis, MO) added to a final concentration of 0.7% was made anaerobic employing alternate cycles of vacuum and pressurization with a mixture of H_2_ and CO_2_ (80:20 v/v, 1.7 × 10^5^ Pa) (Mukhopadhyay et al., 1999). This bottle was then sterilized by autoclaving and brought inside an anaerobic chamber (Coy Laboratory Products, Inc., Grass Lake, MI) quickly enough to prevent solidification of the medium; the anaerobic chamber contained a mixture of N_2_, CO_2_, and H_2_ (76:20:4, v/v/v). The seal of the bottle was removed aseptically, and MgCl_2_, CaCl_2_, Na_2_S, cysteine, and yeast extract were added to the medium from respective anaerobic and sterile stock solutions to final concentrations of 38 mM, 2.45 mM, 2 mM, 2 mM and 0.1%, respectively. Then, the medium was poured onto 100 mm × 15 mm glass petri dishes (VWR, Radnor, PA) and allowed to solidify inside the anaerobic chamber. After inoculation with *M. jannaschii* cells either from a liquid culture or a colony, the plates were placed inside a 2 L steel anaerobic canister (Balch et al., 1979), and a layer of sterile paper towels was placed on the top of the stack of plates. The canister was then closed and pressurized with a mixture of H_2_ and CO_2_ (80:20 v/v) to 3 × 10^5^ Pa. Two milliliter of anaerobic solution of 1 M Na_2_S was added into the canister through a rubber stopper-sealed addition port in a manner that the liquid soaked into the paper towel layer, and the canister with the plates was incubated inside an oven at 80°C. When needed, following components were added into the growth medium: mevinolin (10 and 20 μM for the solid and the liquid media, respectively) and Na_2_SO_3_ (2 and 10 mM). Growth of *M. jannaschii* in liquid culture was followed by measuring the optical density at 600 nm by using Beckman Coulter DU800 spectrophotometer (Brea, CA).

*Methanocaldococcus* strain FS.406-22 was grown in a nitrogen-fixing medium as described previously (Mehta and Baross, 2006). *Escherichia coli* Stellar™, which was used for plasmid constructions, was cultivated in lysogeny broth (LB) or on respective agar plates (Bertani, 2004); the medium was supplemented with ampicillin at a final concentration of 100 μg/ml, as needed.

### DNA Manipulation and Sequencing

*M. jannaschii* genomic DNA was isolated by employing a previously described method (Sambrook and Maniatis, 1989). Briefly, the cell pellet from a 25 ml culture was resuspended in 1 ml of 25 mM potassium phosphate buffer, pH 7, in a 1.5 ml microcentrifuge tube, and this treatment lysed the cells due to osmotic shock. Then to the lysate, an equal volume of a mixture of phenol, chloroform, and isoamyl alcohol (25:24:1, v/v/v) was added, and the mixture was shaken vigorously by hand in the capped tube. The aqueous phase of this mixture was separated from the organic phase by centrifugation at 10,000 ×*g* at 4°C for 10 min and then collected and mixed with an equal volume of a mixture of chloroform and isoamyl alcohol (24:1, v/v). From the aqueous phase of this second mixture, which was retrieved as described above, DNA was precipitated by the addition of an equal volume of isopropanol and one-tenth volume of 3 M sodium acetate buffer, pH 5.3. The precipitated DNA was collected *via* centrifugation at 10,000 ×*g* at 4°C for 10 min, washed with ice-cold 70% ethanol, air dried, and dissolved in 200 μl water. Plasmid DNA was isolated and manipulated following general protocols described previously (Sambrook and Maniatis, 1989); in some cases, spin columns from Qiagen (Venlo, Netherlands) were used for the purification of plasmids from *E. coli* and DNA from PCR reaction mixtures and agarose gels. DNA sequencing was performed at the Genomics Sequencing Center of the Biocomplexity Institute of the Virginia Tech.

DNA hybridization was performed employing DIG-High Prime DNA labeling and detection kit (Roche Applied Sciences, Germany) (Lai et al., 2006). Briefly, the plasmid pDS261 was digested with *Hind*III and *Bam*HI, respectively. The resulting fragments were labeled with digoxigenin (DIG) and were used to probe membrane blots carrying *M. jannaschii* BM31 genomic DNA that was digested with *Pst*I and *Sac*I. The hybridization was performed at 42°C and the hybridizing bands were detected by using alkaline phosphatase-conjugated anti-DIG antibody, 5-bromo-4-chloro-3-indolyl-phosphate (BCIP), and nitro blue tetrazolium (NBT).

### Plasmid Constructions

**Figure 1:**
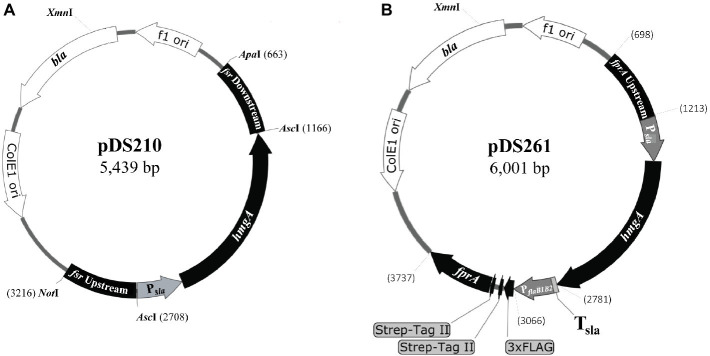
Suicide plasmids used in the construction of a chromosomal deletion and overexpression of a homologous protein with an affinity tag in *M. jannaschii*. The plasmids were pBluescript II SK(+) derivatives carrying a *M. jannaschii*-specific selectable marker composed of the HMG-CoA reductase gene (*hmg*A, *mj_0705*) of *M. jannaschii* (Bult et al., 1996) driven by a putative promoter of the S-layer gene (*mfs40622_1341*) of *Methanocaldococcus* FS.406-22 (P*sla*) (Mehta and Baross, 2006). The marker conferred resistance to mevinolin and simvastatin in *M. jannaschii*. **(A)** The plasmid pDS210, which was used as a suicide plasmid for deleting the *fsr* gene, contained 500 bp long DNA segments of the upstream and downstream regions of the *fsr* gene of *M. jannaschii* (locus tag number, *mj_0870*). **(B)** The plasmid pDS210 was used for constructing a chromosomal construct for the overexpression of an F_420_H_2_ oxidase gene (*fpr*A, locus tag *mj_0748*) with an NH_2_-terminal 3xFLAG-twin Strep affinity tag in *M. jannaschii*. It carried a cassette composed of a putative promoter of the flagellin operon (*flaB1B2, mj_0891-0892*) of *M. jannaschii* (P*_flaB1B2_*) fused with a 174 bp long DNA element encoding the affinity tag which was flanked by 500 bp of the upstream region of *fpr*A (locus tag number, *mj_0748*) and the 500 bp of the 5′ end of *fpr*A coding sequence. *bla*, beta-lactamase gene conferring resistance to penicillin derivatives in *E. coli*. f1 ori and ColE1ori are origins of replication of the f1 phage and ColE1 plasmid, respectively. T_sla_, putative terminator region of S-layer protein gene (*mfs40622_1341*) of *Methanocaldococcus* FS.406-22. Numbers on the side of plasmid maps represent sequence coordinates.

Plasmids used in this study are listed in Table 1 and Figure 1. To construct a suicide plasmid that was used for deleting the *fsr* gene in *M. jannaschii*, two ~500 bp long DNA elements representing respective upstream and downstream regions of *fsr* (locus tag, *mj___0870*) were PCR amplified employing the oligonucleotide primers as listed in Supplementary Table S1 and cloned into *Not*I and *Apa*I sites of pBluescript II SK(+) (Agilent Technologies, Inc., Santa Clara, CA), resulting into pDS200. Then, a linearized form of pDS200 obtained by digestion with *Asc*I that cut the plasmid between the above-mentioned upstream and downstream elements was assembled with two PCR amplicons that carried putative promoter of S-layer protein gene (P*_sla_*) of *Methanocaldococcus* sp. FS-406-22 (*sla*; locus tag: *mfs*40622_1341) (Supplementary Table S2) and the coding region of the HMG-CoA reductase gene of *M. jannaschii* (*hmg*A; locus tag, *mj_0705*) using the In-Fusion^®^ HD cloning kit (Takara Bio, Inc., Mountain View, CA). The assembly generated pDS210 (Figure 1A).

**Table 1:** Plasmids and *M. jannaschii* strains.

Plasmids	Features	Reference
pDS200	A pBluescript II SK(+)-based vector containing 500 bp of the upstream and downstream regions of *fsr* cloned into *Not*I and *Apa*I sites	This study
pDS210	A suicide plasmid for the deletion of *fsr* gene *(mj_0870)*	This study
pDS261	A suicide plasmid for overexpression of FprA protein (*mj_0748*) driven by P*_flaB1B2_* promoter	This study
**Strains**	**Genotypes**	**Reference**
JAL-1	Wild type	(Jones et al., 1983; Mukhopadhyay et al., 1999)
DSM 2661	Type strain	(Jones et al., 1983)
BM10	Δ*fsr::hmgA*	This study
BM10-2661	Δ*fsr::hmgA*	This study
BM31	P*_fprA_*::P_sla_.*hmg*A.T*_sla_*-P*_flaB1B2_*-3xFLAG-2xSTREP	This study

The suicide vector pDS261 that was used for constructing a chromosome-based homologous overexpression system for *M. jannaschii fpr*A (locus tag, *mj___0748*) was developed by assembling the following fragments using In-Fusion^®^ HD cloning kit (fragment, size): pBluescript II SK+ linearized *via* digestion with *EcoR*V, 2,961 bp; upstream region of *fpr*A gene of *M. jannaschii*, 515 bp; P*_sla_* as mentioned above, 315 bp; coding region of *M. jannaschii hmgA*, 1,218 bp; T*_sla_*, putative terminator of the S-layer protein gene of *Methanocaldococcus* sp. FS-406-22 (*sla, mfs40622_1341*), 34 bp; P*_flaB1B2_*, putative promoter of flagellin gene of *Methanocaldococcus jannaschii* (*flaB1, mj_0891*), 282 bp; a synthetic DNA piece encoding 3xFLAG-Twin Strep affinity tag that was codon optimized for expression in *M. jannaschii*, 174 bp (Supplementary Table S1); and 5′ end of *M. jannaschii fpr*A, 501 bp (Supplementary Table S2). Unless mentioned otherwise, a DNA fragment was obtained *via* PCR amplification.

### Transformation of *M. jannaschii*

*M. jannaschii* cells grown in liquid medium as described above but at 65°C were used for transformation. When the culture optical density at 600 nm reached the value of 0.5–0.7, which corresponded to total cell counts of 2–4 × 10^8^/ml as determined microscopically (Mukhopadhyay et al., 2000), the culture bottle was brought inside the anaerobic chamber. The cells were harvested inside the chamber by centrifugation at 3,000 rpm and room temperature for 10 min in an IEC MediSpin Centrifuge (Thermo Electron LED GmbH, Germany). The cell pellet was then resuspended in 500 μl of pre-reduced medium 1 (containing sodium sulfide), and the suspension was treated as follows: (1) incubated at 4°C for 30 min, (2) supplemented with 2 μg of linearized pDS210 or pDS261 generated by digestion with *Xmn*I (Figure 1), (3) incubated at 4°C for an additional hour, (4) subjected to heat shock by incubation at 85°C for 45 s, and (5) incubated at 4°C for 10 min. The resulting mixture was then added to 10 ml pre-reduced medium 1 that was supplemented with yeast extract to a final concentration of 0.1%. The inoculated culture with a mixture of H_2_ and CO_2_ (80:20, v/v; 3 × 10^5^ Pa) in the head space was incubated overnight at 80°C without shaking. One hundred microliter of this culture was plated onto solid medium containing the components necessary for rapid growth and selection of the transformants.

### Overexpression and Affinity Purification of FprA

*M. jannaschii* BM31 expressing FprA under the control of P*_flaB1B2_* was grown in 200 ml liquid medium 1 supplemented with mevinolin to a final concentration 10 μM in a 530 ml serum bottle. When the optical density (OD_600nm_) of the culture reached 0.5–1, which corresponded to actual total cell counts of 2–6 × 10^8^/ml, the cells were harvested aerobically *via* centrifugation at 18,000 ×*g* for 10 min. The resulting cell pellet was stored at −20°C until used.

Purification of FprA was performed under air at 4°C. A cell pellet obtained from 1 L culture (0.8 g wet cell pellet) was resuspended in 2 ml of potassium phosphate buffer (100 mM, pH 7). The cells in this suspension were lysed as described previously (Johnson and Mukhopadhyay, 2005), and the lysate was clarified *via* centrifugation at 18,000 ×*g* for 30 min at 4°C. The supernatant was loaded onto a 1 ml Strep-Tactin^®^ XT column (IBA Lifesciences, Goettingen, Germany) that was previously equilibrated with four bed volumes of wash buffer (100 mM Tris-HCl, pH 8, and 300 mM NaCl). After loading the extract, the column was washed with 4 ml of the wash buffer, the bound proteins were eluted with 4 ml of 10 mM D-biotin solution prepared in wash buffer, and the product was collected in eight fractions of equal volume. The fractions were examined *via* SDS-PAGE and those containing Mj-FprA, as judged by the presence of a 54 kDa polypeptide in it, were pooled.

### Protein Techniques

Protein concentration was determined according to Bradford (Bradford, 1976) by utilizing a dye reagent purchased from Bio-Rad Laboratories, Inc. (Hercules, CA), and SDS-PAGE was performed according to Laemmli (1970). Western blotting was performed as described previously (Mukhopadhyay et al., 1995) with the following modifications. Monoclonal ANTI-FLAG^®^ M2 antibody produced in mouse (catalog number, F1804; Sigma-Aldrich, Inc., St. Louis, MO) served as the primary antibody, and anti-mouse IgG (whole molecule) rabbit antibody conjugated with alkaline phosphatase (catalog number, A2418; Sigma-Aldrich) was the secondary antibody. The antibody reacting band was detected with BCIP and NBT.

### F_420_H_2_ Oxidase Activity Assay

The F_420_H_2_ oxidase activity of purified Mj-FprA was assayed as described previously with modifications (Seedorf et al., 2004). Assays were performed at 70°C anaerobically, and it utilized a rubber stopper-sealed round glass cuvette with N_2_ (1.3 × 10^5^ Pa) in the head space (Daniels and Wessels, 1984; Susanti et al., 2016). The assay began with a 1 ml anaerobic mixture containing the following (component, final concentration): potassium phosphate buffer pH 7, 100 mM; KCl, 100 mM; F_420_H_2_, 40 μM; and 20 nmol oxygen added as 80 μl air saturated water; the concentration of dissolved oxygen in saturated water at room temperature was estimated to be around 250 μM (Seedorf et al., 2004). To this mixture, purified Mj-FprA was added to a final concentration of 10 pM and the F_420_H_2_ oxidase activity was monitored by following the increase in the absorbance of the mixture at 420 nm due to the appearance of F_420_.

### Mass Spectrometry

A preparation of affinity purified Mj-FprA was digested in solution with thermolysin, and the resulting peptide mixture was analyzed by employing an UltiMate™ 3000 RSLCnano system coupled to a Thermo Fusion Orbitrap™ Tribrid™ mass spectrometer (ThermoFisher Scientific, Inc., Waltham, MA). The observed ion masses were used in a Mascot (Matrix Science, Inc., Boston, MA) search against a dedicated database developed for Mj-FprA carrying a NH_2_-terminal 3xFLAG-Twin Strep tag. These analyses were performed at the University of Illinois Protein Sciences Facility (Urbana Champaign, IL).

## Results

### Antibiotic Selection and Selectable Marker for *M. jannaschii*

#### Growth on Solid Medium

The ability to grow a microorganism on a solid medium reproducibly for clonal isolation is a crucial need for its genetic analysis. Accordingly, our first step for the study was to establish the respective method for *M. jannaschii*. We used Gelrite^®^ gellan gum as a gelling agent, which is suitable for work with hyperthermophiles (Lin and Casida, 1984; Grogan, 1989; Childers et al., 1992; Sato et al., 2003). The composition of the solid medium was similar to that of the liquid medium except the former contained cysteine or titanium (III) citrate, at final concentrations of 2 or 0.14 mM, respectively. *M. jannaschii* failed to grow on solid medium lacking the additional reducing agent even though sulfide (2 mM) was provided. To yield larger colonies that are easy to pick, yeast extract was added to the medium and pickable colonies appeared after 2–3 days of incubation.

#### Antibiotic Selection

Like other archaea (Jones et al., 1983; Allers and Mevarech, 2005), *M. jannaschii* was resistant to most antibiotics that are used in the genetic analysis of bacteria. In addition, the growth of *M. jannaschii* was not inhibited by the following antibiotics that are commonly used for work with archaea (entity, concentration): neomycin, 1 mg/ml; puromycin, 250 μg/ml; and novobiocin, 10 μg/ml. This archaeon was also resistant to the base analogs that are used for counter selection in archaea such as 6-methylpurine (0.25 mg/ml), 6-thioguanine (0.25 mg/ml), 6-azauracil (0.75 mg/ml), 5-fluorouracil (0.25 mg/ml), 8-azahypoxanthine (0.25 mg/ml), and 8-aza-2,6-diaminopurine (0.4 mg/ml). However, *M. jannaschii* was found to be sensitive to mevinolin and simvastatin. The concentrations of mevinolin required to fully inhibit the growth in liquid and solid media were 20 and 10 μM, respectively, and the corresponding value for simvastatin is 10 μM (tested only in liquid culture). Mevinolin and simvastatin are competitive inhibitors of 3-hydroxy-methylglutaryl (HMG)-CoA reductase (HMGR), the rate-limiting enzyme in the mevalonate pathway for isoprenoid synthesis in Archaea (Matsumi et al., 2011). Isoprenoids are building blocks of archaeal membrane lipids (Matsumi et al., 2011). Inhibitors of HMGR have been used as selecting agents for genetic manipulations of halophilic archaea such as *Haloferax volcanii* and thermophilic archaea such as *Pyrococcus furiosus, Sulfolobus solfataricus*, and *Thermococcus kodakarensis* (Lam and Doolittle, 1992; Aagaard et al., 1996; Sato et al., 2003; Matsumi et al., 2007; Waege et al., 2010; Lipscomb et al., 2011; Zhang and Whitaker, 2012; Zheng et al., 2012). Since it was available in the laboratory and we determined that it retains its inhibitory activity on wild-type *M. jannaschii* even after incubation at 80°C in growth medium for 5 days, mevinolin was used for most of studies described below. Simvastatin was used in a confirmatory test conducted at the later stage of the study.

#### Overexpressed HMG-CoA Reductase Gene (*hmgA*) as a Selectable Marker

A previous study on the characterization of naturally occurring mevinolin-resistant strains of *Haloferax volcanii* revealed two types of genomic changes that caused this phenotype: generation of multiple copies of *hmgA* and a single base mutation (G →T) in the promoter region of *hmgA* (Lam and Doolittle, 1992). Both changes lead to increased expression of HMGR (Lam and Doolittle, 1992). Guided by this precedence, in this study the overexpression of HMGR was selected as an avenue for imparting resistance to mevinolin in *M. jannaschii* and we decided to use a strong constitutive promoter for this purpose. *M. jannaschii* genome encodes a single *hmgA* gene (*mj_0705*) (Bult et al., 1996).

**Figure 2:**
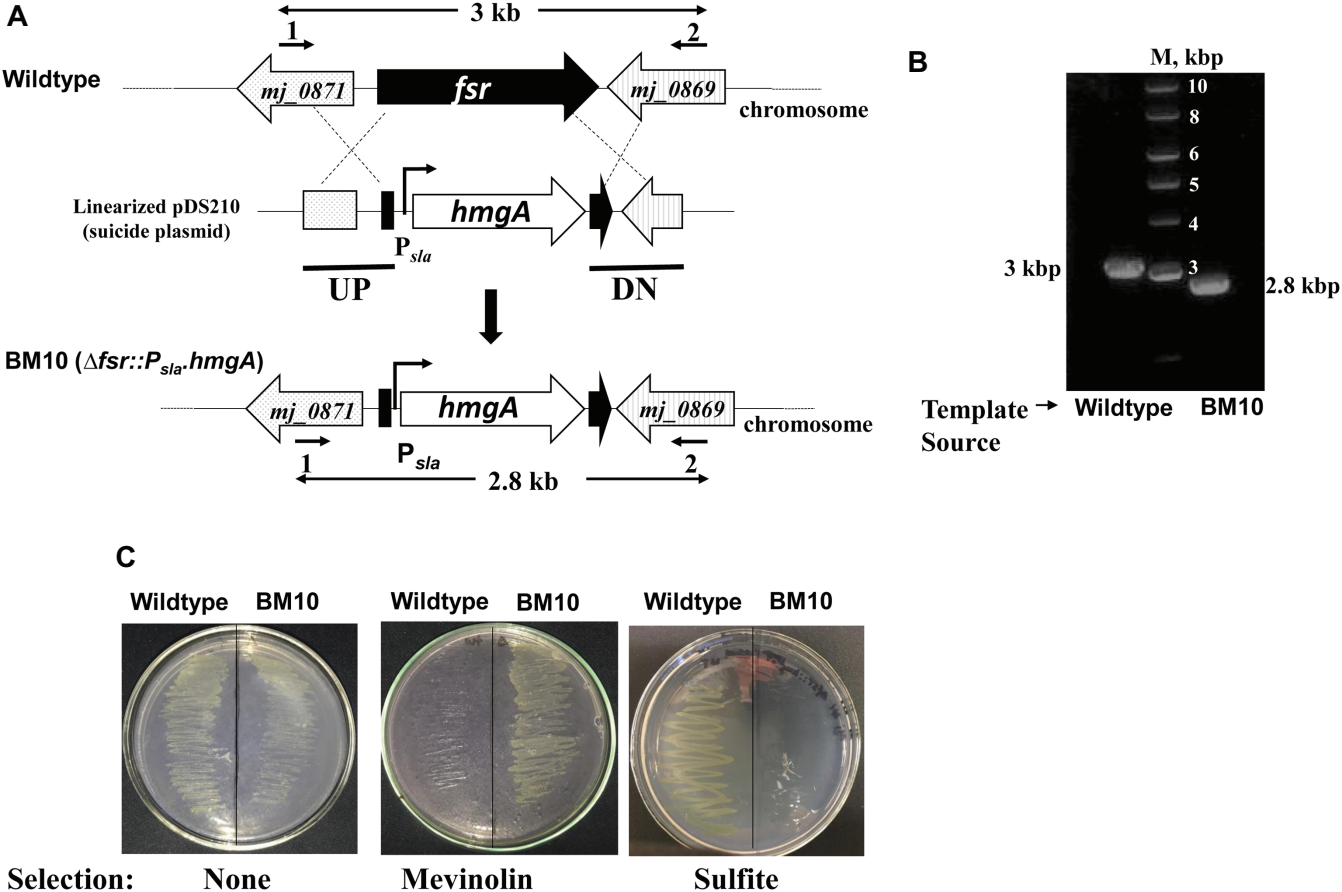
A system for constructing chromosomal deletion in *M. jannaschii* employing the Hmg-CoA reductase gene (*hmgA*) as a selectable marker. Construction of a *M. jannaschii* BM10 (Δ*fsr::P_sla_.hmgA*) strain. **(A)** Replacement of *fsr via* double cross-over recombination between the upstream (UP) and downstream (DN) regions of *fsr* (locus tag number, *mj_0870*) and cloned homologous elements in a linearized form of the suicide plasmid, pDS210 (Figure 1A). **(B)** Characterization of *M. jannaschii* BM10 genotype by the use of PCR analysis. PCR was performed on isolated chromosomal DNA of *M. jannaschii* BM10 employing primers 1 and 2 as shown in A; primer sequences appear in Supplementary Table S1. The genomic regions targeted for PCR amplification are shown with bold double-sided arrows in **(A)**. Sizes of DNA markers (M) and the PCR amplicons are shown next to the respective DNA bands. **(C)** Growth phenotypes of *M. jannaschii* wild-type and BM10 strains on Gelrite plates under three conditions: without selection, under mevinolin (10 μM) selection, and in the presence of 10 mM sulfite. Details of some of the abbreviations and pDS210 appear in the legend of Figure 1.

**Figure 3:**
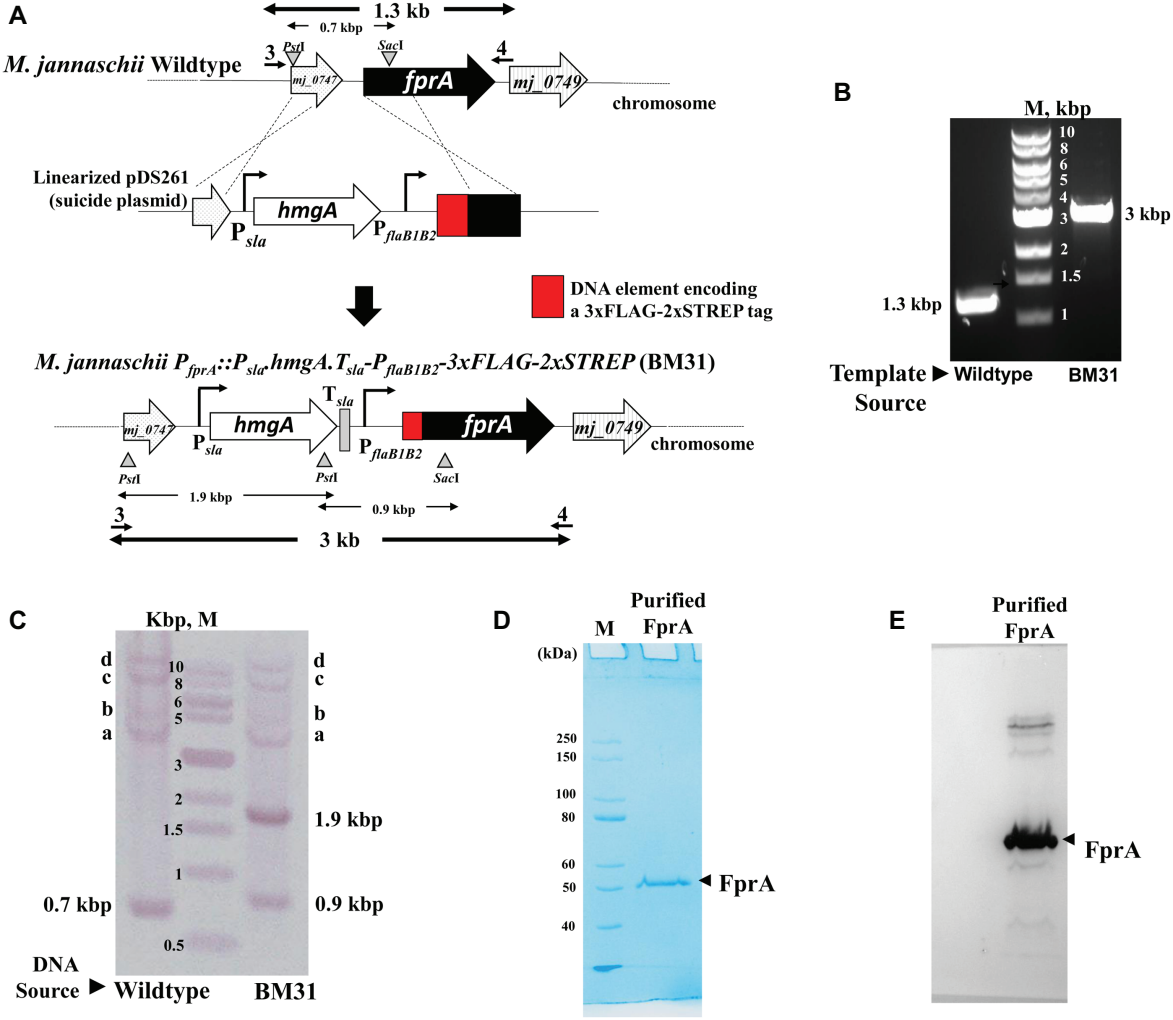
Homologous expression of an affinity tagged protein driven by the *fla*B1B2 promoter in *M. jannaschii*. **(A)** Construction of *M. jannaschii* strain BM31 (P*_fprA_*::P*_sla_.hmg*A.T_sla_.P*_flaB1B2_*-3xFLAG-Twin-Strep.*fpr*A) expressing F_420_H_2_ oxidase (FprA) protein under the control of P*_flaB1B2_*, the promoter for the flagellin (*fla*B1B2) operon of *M. jannaschii*. The strain was constructed *via* double cross-over recombination between the upstream and coding regions of *fpr*A (locus tag number, *mj_0748*) of the *M. jannaschii* chromosome and cloned homologous elements in a linearized form of pDS261, a suicide vector (Figure 1B). **(B)** PCR analysis of the genotype of *M. jannaschii* BM31. Primers 3 and 4, as shown in **(A)**, were used for the amplification, and the respective sequences appear in Supplementary Table S1. The genomic regions targeted for PCR amplification are shown with bold double-sided arrows in **(A). (C)**. Southern DNA hybridization analysis. Genomic DNA samples of wild-type and BM31 strains of *M. jannaschii* were digested with *Pst*I and *Sac*I. The suicide plasmid pDS261 was digested with *Hind*III and *BamH*I. The relevant restriction enzyme sites on the genome and suicide plasmid and the corresponding DNA fragments are shown with triangles/inverted triangles and double-sided arrows, respectively, in **(A)**. A mixture of fragments resulting from the restriction enzyme digestion of pDS261 were labeled with Digoxigenin and used as hybridization probe. Observed hybridizing band, respective identity: 0.7, 0.9, and 1.9 kbp, shown in **(A)**; a–c, P_*fla*B1_, *fpr*A, and *hmg*A regions of *M. jannaschii* chromosome as shown in Supplementary Figure S2; d, partially digested high molecular mass DNA that hybridized with the DIG-labeled probes. **(D)** An SDS-PAGE profile of affinity purified Mj-FprA. Purified protein (0.5 μg) was analyzed on a 10% SDS-PAGE gel. The apparent molecular mass of Mj-FprA polypeptide was 54 kDa. M, unstained protein standard (New England Biolabs, Ipswich, MA). The masses of protein markers are shown next to the respective bands. (**E**) Western blot hybridization of Mj-FprA employing monoclonal anti-FLAG^®^ M2 mouse antibody and visualized using an anti-mouse IgG (whole molecule) rabbit antibody conjugated with alkaline phosphatase and chromogenic alkaline phosphatase substrates, NBT and BCIP.

We selected the putative promoter for the S-layer protein gene (P*_sla_*) of *Methanocaldococcus* strain FS.406-22 (locus tag: *mfs*40622_1341), which was available in the laboratory (Dwi Susanti, unpublished information), to drive the expression of *M. jannaschii hmg*A. This promoter was selected for the following reasons. The cell wall of a *Methanocaldococcus* species is composed of a paracrystalline layer made up of the S-layer protein (Boot and Pouwels, 1996). Since it is the building block of a major structural unit of the cell, the S-layer protein is expected to be expressed at a high level in all *Methanocaldococcus* species and it is true for *M. jannaschii* (Eric Johnson and Biswarup Mukhopadhyay, unpublished analysis of a 2D-gel pattern; Mukhopadhyay et al., 2000). This deduction is also consistent with the observation that generally S-layer represents 10–15% of total protein in archaea and bacteria (Boot and Pouwels, 1996). An S-layer promoter has been used for driving gene expression in *Methanococcus voltae* (Thomas et al., 2002). While the P*_sla_* of *Methanocaldococcus* strain FS.406-22 is yet to be characterized, the transcription start site, core promoter element called TATA box, and transcription factor IIB (TFIIB)-recognition element (BRE) of the *M. jannaschii sla* gene are known (Zhang et al., 2009; Smollett et al., 2017). We found that the TATA and BRE elements of the *M. jannaschii sla* gene were conserved in 5′-untranslated region (5′-UTR) of the *Methanocaldococcus* strain FS.406-22 *sla* gene. Using this similarity as a guide, a 316 bp DNA element that contained the translation start codon and the immediate upstream region bearing the putative TATA and BRE elements of the *Methanocaldococcus* strain FS.406-22 *sla* gene was selected to serve as P*_sla_* (Supplementary Figure S1). This P*_sla_* element was fused with the coding sequence of *M. jannaschii hmgA* to generate an antibiotic resistance gene cassette (P*_sla_-hmgA*) for use as a selectable marker (Figures 1, 2A, 3A; Supplementary Table S1).

### A Gene-Deletion System in *M. jannaschii*

#### Target Gene and Suicide Plasmid

We selected the F_420_-dependent sulfite reductase (Fsr) gene (*fsr*) (Johnson and Mukhopadhyay, 2005) as a target for testing the utility of the selection agent (mevinolin) and a cognate selectable marker (P*_sla_-hmgA* cassette) as described above for constructing a gene knockout in *M. jannaschii*. Fsr catalyzes 6-electron reduction of sulfite to sulfide (Johnson and Mukhopadhyay, 2005). It is a unique enzyme as it uses F_420_H_2_, a reduced form of coenzyme F_420_, as an electron donor instead of the most common reductants for sulfite reductases, namely NADPH or ferredoxin (Johnson and Mukhopadhyay, 2005).

Structurally, Mj-Fsr belongs to the group of dissimilatory sulfite reductases (Johnson and Mukhopadhyay, 2005). However, in *M. jannaschii*, it functions as a sulfite detoxification and assimilatory enzyme, converting toxic sulfite oxyanion to sulfide, which is a sulfur source and essential nutrient for *M. jannaschii* (Johnson and Mukhopadhyay, 2005). *M. maripaludis*, which is sensitive to sulfite, becomes proficient in not only tolerating sulfite but also using it as sole sulfur source when *M. jannaschii fsr* is expressed recombinantly in this organism (Johnson and Mukhopadhyay, 2008). Thus, it is likely that *fsr* is the key determinant of sulfite reduction ability in *M. jannaschii*.

This protein is produced in *M. jannaschii* only when the growth medium is supplemented with sulfite (Johnson and Mukhopadhyay, 2005), and therefore, it is unlikely to be essential for the growth of this archaeon with sulfide as sulfur source. Consequently, the deletion of *fsr* in *M. jannaschii* would result in a viable mutant strain with a clear phenotype, which would be a sensitivity to sulfite. These possibilities made *fsr* a good target for developing a method for constructing a gene knockout in *M. jannaschii.*

For the purpose mentioned above, we developed pDS210 (Figure 1A), a suicide plasmid that would replicate in *E. coli* but unlikely in *M. jannaschii.* It was based on pBluescript II SK(+), a high copy *E. coli* plasmid, and carried the P*_sla_-hmgA* cassette flanked by two DNA elements representing the upstream and downstream regions of *fsr* (Figure 1A). Considering that the terminator of the *fsr* gene will allow transcription termination function for the inserted *hmgA*, we did not introduce an additional termination in the selectable marker construct (Figure 1A).

#### Transformation and Selection of Transformants

To generate *M. jannaschii* cells that would be more proficient in taking up DNA, we opted for growth at 65°C based on the following logic. *M. jannaschii* membrane is composed of ether-linked lipids that are made of isoprenoid chains with various configurations and degrees of unsaturation (Sprott, 1992). Of these lipids, the tetraethers that span the entire width of the membrane likely impart the highest degree of rigidity of all ether-linked lipids, and the macrocyclic diethers where a single isoprenoid chain contributes to both ether linkages of an archaeal lipid are more rigid than simple diethers (Sprott, 1992). It has been shown that as growth temperature increases the membrane of *M. jannaschii* gets enriched in the tetraethers and macrocyclic diethers, and this change is consistent with a need to maintain membrane integrity at higher temperatures (Ferrante et al., 1990). For this fact, we reasoned that the membrane of a *M. jannaschii* cell grown at 65°C would be more permissive to the entry of DNA, and at this temperature, the organism exhibits reasonably fast growth; the observed generation time at 65 and 85°C are 111 and 26 min, respectively (Jones et al., 1983).

To avoid the integration of the entire suicide plasmid into the chromosome that would generate merodiploid and consequently sulfite tolerant cells, we used a linearized form of pDS210 for transformation; the linearized form was generated *via Xmn*I digestion (Figure 1A). We have attempted the DNA delivery *via* heat shock, which is effective in transforming of *Methanococcus voltae, Thermococcus kodakarensis, Pyrococcus furiosus*, and *Sulfolobus acidocaldarius* (Bertani and Baresi, 1987; Aagaard et al., 1996; Sato et al., 2003; Lipscomb et al., 2011). In one trial, cells were treated with CaCl_2_ prior to mixing with DNA and subjecting to heat shock and in the other without CaCl_2_ treatment. The treated cells were plated on solidified medium containing mevinolin at a concentration of 10 μM. Both methods produced equivalent numbers of colonies on the plates. We optimized the latter method, and it is described in detail in the section Materials and Methods. This procedure did not produce colonies on the above-mentioned mevinolin-Gelrite^®^ plates if pDS210 was omitted from the transformation mixture. The mevinolin-resistant strain obtained from the transformation was named *M. jannaschii* BM10. The results presented in Figure 2C show that *M. jannaschii* BM10, but not the wild-type strain, was resistant to mevinolin. Typically, 10^4^ mevinolin-resistant colonies were observed from transformation with 1 μg of pDS210 DNA. We performed a gene knockout experiment with *M. jannaschii* DSM 2661 employing pDS210 and obtained 5 × 10^3^ mevinolin-resistant colonies per microgram plasmid DNA; we call the resulting strain *M. jannaschii* BM10-2661.

Mevinolin and simvastatin are structurally related, and as mentioned above, both compounds inhibit 3-hydroxy-methylglutaryl (HMG)-CoA reductase. Accordingly, we tested the sensitivity of *M. jannaschii* BM10 to simvastatin. We found that in liquid medium BM10 was resistant to simvastatin supplied at a concentration of 10 μM, while the wild type was inhibited by this antibiotic. These results indicated that while the minimum inhibitory concentration of this compound that is needed to inhibit the growth of the organism on solid medium is yet to be determined, simvastatin could safely be considered as a substitute of mevinolin for selecting *M. jannaschii* strains bearing the P*_sla_-hmgA* cassette.

#### Genotypic Characterization of *M. jannaschii Δfsr* Strain

The mevinolin-resistant *M. jannaschii* BM10 was genotypically characterized employing PCR-based analysis of genomic DNA. This analysis considered a double recombination process (Figure 2A) that led to the formation of *M. jannaschii* BM10. The sizes of PCR amplicons obtained with genomic DNA of BM10 and wild-type strains of *M. jannaschii* as templates and the primers 1 and 2 as shown in Figure 2A and Supplementary Table S1, and the respective determined sequences matched the expectations (Figure 2B). These results showed that BM10 was generated by the replacement of *fsr* coding region with *P_sla_-hmgA* cassette and described the genotype of the strain as *Δfsr::P_sla_-hmgA* (Figure 2A). The same result was obtained with *M. jannaschii* BM10-2661 (data not shown).

#### Phenotypic Characterization of *M. jannaschii* BM10

**Figure 4:**
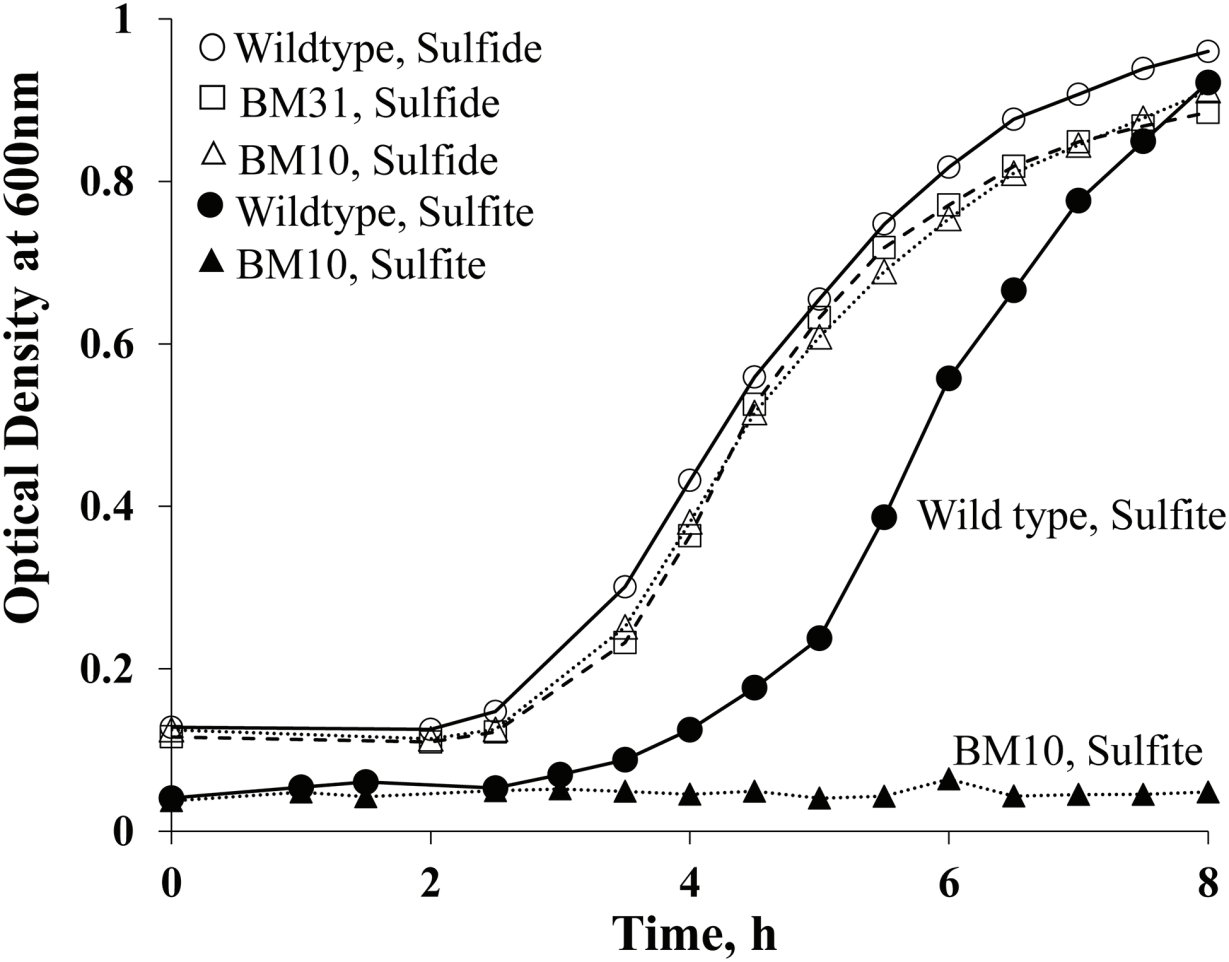
Growth phenotypes of *M. jannaschii* strains grown with sulfide or sulfite as medium reductant and sulfur source. The study was performed as described previously (Mukhopadhyay et al., 1999; Johnson and Mukhopadhyay, 2005) using 530 ml sealed serum bottles, each containing 100 ml medium and a mixture of H_2_ and CO_2_ (80:20, v/v) in the head space at a pressure of 3 × 10^5^ Pa (Mukhopadhyay et al., 1999; Johnson and Mukhopadhyay, 2005). Sulfite or sulfide concentration, 2 mM. When sulfite was used as sulfur source, after sulfite addition but prior to inoculation, the medium was pre-incubated at 80°C for 14 h to achieve adequate reduction as evidence by the clearing of the color of resazurin; resazurin is a redox indicator commonly used in methanogen media (Balch et al., 1979). Pre-warmed and pre-reduced media were inoculated with 2 ml of an overnight culture developed under the test condition, with one exception. For testing the ability of BM10 to grow with sulfite as sulfur source, the inoculum was developed with sulfide. Each reported optical density value is an average from measurements with two independent cultures.

We compared the growth pattern of the BM10 or *Δfsr::P_sla_-hmgA* strain of *M. jannaschii* with that of the wild type both in liquid and on solid media. In liquid medium with sulfide (2 mM) as medium reductant and sulfur source, these strains had comparable growth phenotypes (Figure 4). However, with sulfite (2 mM) as the sole sulfur source and medium reductant, BM10 failed to grow (Figure 3), and the same was the case when the liquid medium contained both sulfide and sulfite, each at 2 mM concentration. *M. jannaschii* BM10 exhibited a different phenotype when it was grown on a solid medium, which contained yeast extract (1 g/L), titanium (III) citrate (0.14 mM), and Gelrite^®^ (7 g/L). It formed colonies in the presence of sulfite at a concentration of 2 mM but not 10 mM. It should be noted that the plates (total number, 8) were incubated in a jar that contained paper towels soaked with 2 ml of 1 M sodium sulfide solution to provide a reduced environment. This set up would have provided at the most a sulfide concentration of 20 mM in the solid medium, assuming all of the sulfide was trapped in the medium. Wild-type *M. jannaschii* grew on plates containing either 2 or 10 mM sulfite under these conditions. While the physiological basis for this difference between the growth phenotypes observed in liquid and on solid media is currently unknown, the reported observation would be an important factor in strain construction efforts where *fsr* will be a selectable marker. The development of *M. jannaschii* colonies on solid medium within 2–3 days requires a medium with complex composition and highly reduced environment that have been described in this report. Under these conditions, selection of a transformant developed from the *Δfsr* or BM10 as a parent strain with a *fsr* cassette as selectable marker would require the use of sulfite at a concentration of 10 mM and not 2 mM, as the latter concentration would not suppress the growth of BM10; we are currently conducting experiments to determine the minimum effective sulfite concentration which would lie between 2 and 10 mM.

### A System for Engineering the Overexpression of a Protein With an Affinity Tag in *M. jannaschii*

#### Promoter for Protein Overexpression

With the availability of the above described tools to modify the genome of *M. jannaschii*, we attempted to develop a method to engineer a chromosome-based system for overexpression of a protein with an affinity tag in this organism. To drive the transcription for this purpose, we selected the putative promoter of the *flaB1B2* (P*_flaB1B2_*) operon of *M. jannaschii*. The *flaB1B2* operon encodes the flagellins FlaB1 and FlaB2, and these are expressed at elevated levels when *M. jannaschii* experiences a low hydrogen partial pressure (Mukhopadhyay et al., 2000). An analysis of a previously published 2D-gel image (Mukhopadhyay et al., 2000) suggested that FlaB1 and FlaB2 together constitute about 12.4% of the total protein of a *M. jannaschii* cell. Also, in a sealed serum bottle system that is commonly used for the cultivation of *M. jannaschii* in the laboratory, the organism experiences hydrogen limitation as growth progresses, which in turn promotes the production of flagellins (Dwi Susanti and Biswarup Mukhopadhyay, unpublished data); *M. jannaschii* cells without flagella are seen in a bioreactor-based culture constantly supplied with hydrogen at a high partial pressure (Mukhopadhyay et al., 2000). Thus, P*_flaB1B2_* was considered a good candidate promoter for engineering a protein expression system that is be suitable for a sealed serum bottle-based cultivation system. The promoter element was designed based on the published information on the transcription start site and promoter of *M. jannaschii flaB1B2* operon and bioinformatic analysis of the 5′-UTR of *flaB1B2* (Thomas and Jarrell, 2001; Zhang et al., 2009; Smollett et al., 2017). It contained the translation start codon of *flaB1*, the BRE-TATA sequence of *flaB1B2*, and an additional 147 bp of the further upstream region (Supplementary Figure S1).

#### F_420_H_2_ Oxidase (FprA) as a Candidate for Overexpression

The FprA of *Methanobrevibacter arboriphilus* or Mar-FprA has been characterized, and it contains FMN and a binuclear iron center (Seedorf et al., 2004). It catalyzes the reduction of O_2_ to H_2_O employing F_420_H_2_ as reductant, and accordingly, it is considered an O_2_ detoxification enzyme for this methanogen (Seedorf et al., 2004). The enzymatic activity of Mar-FprA is oxygen stable, assayed employing a simple protocol and involves coenzyme F_420_ (Seedorf et al., 2004), which is a key coenzyme in methanogens (DiMarco et al., 1990). Thus, it was considered a relevant protein with several structural complexities yet amenable to manipulation under air and suitable for our method development exercise. *M. jannaschii* genome carries two homologs of FprA, Mj_0732 and Mj_0748, and both the primary structure alignment and phylogenetic analysis have described the latter as a closer relative of Mar-FprA (Seedorf et al., 2004). A similar relationship is seen with the FprA from *Methanothermobacter marburgensis* (Mmar-FprA) (Seedorf et al., 2004) for which an X-ray crystallographic structure has been determined (Seedorf et al., 2007). The amino acid sequence identities and similarities of Mj_0748 to both Mar-FprA and Mmar-FprA are 67 and 82%, respectively. In contrast, the corresponding values for Mj_0732 are 40 and 58% with Mar-FprA and 40 and 60% with Mmar-FprA. Also, a global transcriptional analysis has revealed that the *mj_0748* gene is transcribed to a monocistronic mRNA, whereas *mj_0732* is part of a three gene operon that is transcribed into a polycistronic mRNA (Smollett et al., 2017). Consequently, we concluded that we had a better chance of demonstrating F_420_H_2_ oxidase activity in a purified preparation of Mj_0748 than that of Mj_0732. Accordingly, Mj_0748 was chosen as target for this study and is called here as Mj-FprA.

#### Vector and Strain Construction

To develop a *M. jannaschii* strain that would overexpress Mj-FprA with an affinity tag, the suicide plasmid pDS261 was constructed (Figures 1B, 3A). The plasmid contained DNA elements representing the upstream and 5′-end of the coding regions of *mj_0748* that were to allow double cross-over homologous recombination between linearized pDS261 and the chromosome (Figure 3A). This recombination event was designed to couple the 5′-end of *mj_0748* coding region with a 3xFLAG-twin Strep tag coding sequence and place the modified gene under the control of an engineered version of P*_flaB1B2_* (Figure 3A). It was also to introduce the above described P*_sla_-hmgA* cassette, followed by a transcription terminator element (T_sla_), to the 5′-end of the engineered P*_flaB1B2_* unit as a selectable marker. The T_sla_ element represents the putative transcription terminator element of the S-layer protein gene (*orf msf40622_1341*) of *Methanocaldococcus* FS.406-22 (Supplementary Figure S1), and it was positioned to shield the engineered P*_flaB1B2_* from the influence of the P*_sla_* that would be located upstream of the former.

Transformation of *M. jannaschii* with linearized pDS261 provided a mevinolin-resistant strain, named *M. jannaschii* BM31, and the results of a PCR-based analysis of respective chromosomal DNA (Figure 3B) showed that it had the expected genotype, P*_fprA_*::P*_sla_-hmgA-*T*_sla_*-P*_flaB1B2_*-3xFLAG-twin-Strep (Figure 3A). The amplicon sizes and sequences were characteristic of the expected genotype. Since pDS261 is a more complex construct than pDS210, we used this part of the study as a platform to test whether the suicide vector system that we used had the propensity of creating off-target changes in the chromosome *via* homologous recombination with *hmgA* and promoter and terminator elements as well as through illegitimate recombination with pBluescript II SK(+). We performed a Southern blot analysis for this purpose (Figure 3C). The appearance of the characteristic bands as marked with the expected sizes in Figure 3C clearly demonstrated that a double cross-over recombination with the linearized pDS261 provided the desired genotype (Figure 3A) and the backbone of pDS261 did not integrate into the chromosome of *M. jannaschii* (Figure 3C). The bands a–c in Figure 3C were due to the hybridization of parts of the pDS261 with the native *hmgA*, P*_flaB1B2_*, and *fprA* locus of the organism as shown in Supplementary Figure S2; the top most band (band d) that was seen with both the wild-type and BM31 strain was due to partially digested high molecular mass DNA. In terms of growth rate and final culture density in a standard medium, this *fprA* overexpression strain was comparable to the wild-type *M. jannaschii* (Figure 4).

#### Purification and Characterization of Mj-FprA Produced With an Affinity Tag in *M. jannaschii*

The protein was purified from *M. jannaschii* BM31 by the use of a Streptactin XT superflow column from where it was eluted with 10 mM D-biotin. The yield was 0.26 mg purified protein per liter culture. An SDS-PAGE analysis showed that the preparation was homogeneous (Figure 3C). A Western blot analysis using a monoclonal anti-FLAG^®^ M2 mouse antibody (Sigma-Aldrich, St. Louis, MO) showed that the purified protein carried the FLAG-tag (Figure 3D). A mass spectrometric analysis with a thermolysin digest identified 41 peptides that belonged to Mj-FprA and accounted for 55% of the primary structure of the protein including one of the three FLAG tags and the twin Strep tag (Supplementary Figure S3). All these data taken together indicated that *M. jannaschii* was successfully engineered to overexpressed FprA homologously with an affinity tag, which provided a facile purification method.

The purified Mj-FprA protein was tested for the predicted activity by measuring the oxygen reduction activity employing F_420_H_2_ as the reductant as described previously (Seedorf et al., 2004). The apparent specific activity of Mj-FprA at 70°C with oxygen and F_420_H_2_ at concentrations of 20 and 40 μM, respectively, was 2,100 μmole/min/mg. This value was 38 and 19 times higher than that of native FprA of *Methanobrevibacter arboriphilus* and recombinant *Methanothermobacter marburgensis* FprA generated in *E. coli* (Seedorf et al., 2004).

## Discussion

This is the first report of a genetic manipulation of a hyperthermophilic methanogen as well as of a deep-sea hydrothermal vent dwelling methanogen. It describes two new technical capabilities, one of which concerns the construction of designed changes in the genome and the other homologous overexpression of proteins with affinity tags. These tools will facilitate *in vivo* gene function analysis, which in turn will provide physiological relevance to a broad range of studies for which *M. jannaschii* has been a model system.

The genetic manipulation method described here is simpler and less time-consuming than those which are in use for work with other methanogens. The transformation of *M. jannaschii* required a heat shock and not a treatment with a chemical such as polyethylene glycol and liposomes, which are used for work with *M. maripaludis* and *Methanosarcina* species, respectively (Metcalf et al., 1997; Tumbula and Whitman, 1999); liposomes are relatively expensive. Starting from a liquid culture, colonies of *M. jannaschii* strains BM10 and BM31 were generated on a solid medium in 3–4 days. Similar outcomes with *M. maripaludis* and *Methanosarcina* species take ~7 and ~14 days, respectively (Buan et al., 2011; Sarmiento et al., 2011). This advantage in part was due to the fact that *M. jannaschii* grows fast with a doubling time of 26 min (Jones et al., 1983), and the respective values for *M. maripaludis* and *Methanosarcina acetivorans* are 2 and 8.5 h, respectively (Whitman et al., 1986; Oelgeschlager and Rother, 2009). We used linear forms of suicide vectors for constructing desired genome modifications. This method was selected to avoid integration of an entire vector into the chromosome through a single cross-over recombination event and consequent formation of a merodiploid cell. For studies dealing with potentially essential genes or for certain methods of constructing markerless modifications (Pritchett et al., 2004; Moore and Leigh, 2005; Lipscomb et al., 2011; Gehring et al., 2017), it would be necessary to integrate the suicide vectors, and it remains to be determined whether genome engineering with circular substrates would be workable with *M. jannaschii*.

The combination of mevinolin and P*_sla_-hmgA* cassette provided a background-free system for selecting *M. jannaschii* transformants. Considering that the transformants resulted from a double cross-over recombination event, the observed average value of 10^4^ for the colony forming units (cfu) per microgram DNA was comparable or better than those recorded for other hyperthermophilic archaea; the respective values for *Sulfolobus* sp., *Thermococcus kodakarensis, Pyrococcus furiosus* COM1, *Pyrococcus furiosus* DSM 3638, and *Pyrococcus abyssi* are 10^3^–10^6^, 10^2^–10^5^, 10^2^–10^5^, 5 × 10^2^, and 10^2^–10^3^ cfu/μg DNA, respectively (Farkas et al., 2013).

The P*_flaB1B2_*-driven expression system generated a protein of interest with a well-established dual-affinity tag (Gloeckner et al., 2009) and at a relatively high expression level in *M. jannaschii* (Figure 3D). The engineered tag allowed facile detection of the protein *via* an immunological method and purification *via* affinity chromatography (Figures 3D,E). The yield of purified Mj-FprA from engineered *M. jannaschii* cells (0.3 mg/g wet cell) was threefold higher than that for *Methanothermobacter marburgensis* FprA from recombinant *E. coli* (0.1 mg/g of wet cell).

The results from work with the 3xFLAG-Twin Strep-FprA showed that *M. jannaschii* has a *bona fide* process of neutralizing oxygen, an important ability for a deep-sea hydrothermal vent organism. The vent fluid with a temperature of 300–350°C is cooled through a mixing with cold oxygen containing seawater that permeates through the chimney wall to a stage where an organism such as *M. jannaschii* could grow (Jannasch, 1989). Although the oxygen from seawater is neutralized to a great extent by the sulfide of the vent fluid (Jannasch, 1989), the mixing event could expose a vent organism to oxygen.

The new tools described in this report would facilitate a variety of other types of *in vivo* physiological and structure-function analysis. The process described in Figure 3 technically could be used to manipulate any DNA element in *M. jannaschii*. A 3xFLAG-Twin Strep affinity tag-based tandem affinity purification (TAP) method would allow the isolation of protein complexes with a reduced chance of copurifying functionally unrelated proteins (Gloeckner et al., 2009). The homologous expression system would be useful for work with proteins that utilize *M. jannaschii*-specific prosthetic groups or cofactors, undergo specific types of posttranslational modifications, or require folding by a chaperone from this organism for exhibiting the native activity. An example of such a protein is methyl-coenzyme M reductase with bound coenzyme F_430_ that carries organism-specific structural modifications (Allen et al., 2014). This protein expression system could also be used to generate proteins from other hyperthermophiles in *M. jannaschii* in properly folded forms.

The P*_flaB1B2_*- and P*_fsr_-*driven expression systems have the potentials of being a valuable tool for determining if a gene is essential under a specific growth condition; P*_fsr_* and P*_flaB1B2_* are the promoters of the *fsr* gene and *flaB1B2* operon, respectively. These assumptions are based on the following observations and deductions. *M. jannaschii* synthesizes Fsr protein when the organism is exposed to sulfite (Johnson and Mukhopadhyay, 2005). Consequently, having an essential gene under the control of P*_fsr_* would prevent the growth of *M. jannaschii* unless sulfite is present; the reverse would be true for a gene with a deleterious activity. The rationale for the use of P*_flaB1B2_* is the following. *M. jannaschii* grows best under a high hydrogen partial pressure (*p*H_2_) such as ~200 kPa, and under this condition, the flagella synthesis stops (Mukhopadhyay et al., 2000). It is possible that under this hydrogen sufficient condition P*_flaB1B2_* becomes inactive. Consequently, having a gene under the control of P*_flaB1B2_* could prevent its expression if the growth occurs under high *p*H_2_. Such an experiment would be performed most effectively in a bioreactor-based system where a hydrogen sufficient condition could be maintained continually (Mukhopadhyay et al., 2000). However, for both the P*_flaB1B2_*- and P*_fsr_-*driven systems, the proper designs of the desired control elements would require a clear knowledge of the mechanisms underlying observed regulations, which could involve controls at the level of transcription or translation or both. The measurements that brought the *M. jannaschii flaB1B2* and *fsr* systems in focus occurred only at the protein level (Mukhopadhyay et al., 2000; Johnson and Mukhopadhyay, 2005)

Our *M. jannaschii* culture has been maintained either in an active form or as a stock at −80°C. It is possible that under these conditions the organism’s genome has undergone changes, which in turn have made the organism amenable to DNA transformation and/or prone to homologous recombination-driven genome modification. Such an event would be less likely for a stock maintained in liquid nitrogen vapor at a repository or culture collection such as the DSMZ. Considering this possibility and a previously reported experience with *Pyrococcus furiosus* (Lipscomb et al., 2011; Bridger et al., 2012; Farkas et al., 2012), we have examined the transformability of *M. jannaschii* DSM 2661, the type strain, that we obtained from DSMZ. We found that *M. jannaschii* DSM 2661 chromosome could be modified with ease *via* homologous recombination with DNA purified from *E. coli* and delivered into the cell *via* heat shock, and the observed efficiency for this process was about half of that seen with our laboratory strain. This difference in the efficiencies of constructing a chromosomal change in two *M. jannaschii* strains is of minor magnitude compared to above cited finding with *P. furiosus.* In the latter case, the wild-type strain is refractory to chromosome modification *via* homologous recombination with exogenous DNA; however, this process is highly efficient with a laboratory-derived variant that is naturally competent in DNA uptake (Lipscomb et al., 2011; Bridger et al., 2012; Farkas et al., 2012). Thus, the methods and tools described in this report will have wider applicability in work with either our laboratory stock or that available from DSMZ. We plan to determine the whole genome sequence for our laboratory stock, and if the observed genotype is found to be different from that of the type strain, we will deposit our stock to the DSMZ and American Type Culture Collection (Manassas, VA); the new genome sequence will be deposited to the GenBank^®^ (Benson et al., 2013).

The genetic analysis system described in this report could be expanded in several ways. One of these could occur immediately and it concerns the replacement of mevinolin, which has served well thus far but is an expensive reagent. This burden could be lessened by using *M. jannaschii Δfsr::P_sla_-hmgA* as a working strain and *fsr* as a selectable marker, as the transformants but not the *Δfsr* strain will be resistant to sulfite at a concentration of 10 mM; a sulfite salt is substantially less expensive than mevinolin.

The longer-term developments would focus on tools for constructing multi-gene knockouts, additional regulated promoters, and one or more shuttle vectors. Due to the hurdles of designing an effective selection system for *M. jannaschii*, a markerless system is considered a better option for constructing multi-gene knockouts. It could either follow a process that involves the generation of a merodiploid cell with a selectable marker and its segregation to mutant and wild-type forms (Pritchett et al., 2004; Moore and Leigh, 2005; Lipscomb et al., 2011) or employ a flippase (FLP) recombinase for the removal of the selectable marker (Schweizer, 2003); for the latter, a FLP recombinase from a hyperthermophile such as *Sulfolobus shibatae* could be used (Letzelter et al., 2004). Both of these approaches will need the assistance of a counter-selection system (Schweizer, 2003; Pritchett et al., 2004; Moore and Leigh, 2005; Lipscomb et al., 2011). Considering that *M. jannaschii*, an obligate autotroph, could lack efficient transport systems for commonly used selection agents, one option would be to retest the effectiveness of 8-azahypoxanthine, 8-aza-2,6-diaminopurine, and 6-thioguanine at concentrations higher than that generally used in genetic studies with other archaea and bacteria, because the respective marker gene, *hpt*, exists in *M. jannaschii* (Bult et al., 1996). This effort could also include 5-fluoroorotic acid, as the respective marker, *pyrF*, is also present in *M. jannaschii*. For work with *Methanosarcina acetivorans*, a combination of bromoethanesulfonate, an inhibitor of methyl-coenzyme M reductase, and a mutant strain that cannot transport coenzyme M into the cells has been shown to be effective as a counter-selection system (Zhang et al., 2000), and it has been deployed for generating genomic changes in this methanogen (Guss et al., 2005). This methanogen-specific system has the potential of application in *M. jannaschii.* The promoter for the methyl-coenzyme M reductase operon (P*_mcrB_*) could be used for unregulated gene expression; P*_mcrB_* is routinely used for work with *M. maripaludis* and *Methanosarcina* species (Metcalf et al., 1997; Tumbula and Whitman, 1999; Farkas et al., 2013). *M. jannaschii* carries two plasmids (Bult et al., 1996), which could be leveraged for developing shuttle vectors.

## Significances

The tools and methods described in this report make it possible to carry out *in vivo* analyses of *M. jannaschii* metabolism. This is a key strength for functional genomic studies. The genes with unknown functions in *M. jannaschii* genome currently represent 40% of all genes (NCBI accession number, NC_000909.1), which is lower than the original value of 60% (Bult et al., 1996). However, the leads for the function of genes from comparative biology approaches have become limited, and the situation is worse when one seeks a physiological role of a gene. For example, computational and *in vitro* analysis suggest that *M. jannaschii* activates and reduces sulfate (Lee et al., 2011; Cho, 2013), yet the organism cannot utilize sulfate as sulfur source. A genetic analysis would be the best route to assign the physiologically valid roles to these genes. It will also enable more precise analysis of the eukaryotic type DNA replication, transcription, translation, protein translocation and stress management systems, ancient redox control mechanisms, and novel enzymes of *M. jannaschii* (Olsen and Woese, 1996; Koonin et al., 1997; Mukhopadhyay et al., 2000; Johnson and Mukhopadhyay, 2005; Susanti and Mukhopadhyay, 2012; Susanti et al., 2014, 2016) and *in vivo* validation of the biosynthesis pathways that have been derived from studies *in vitro* or in surrogate systems (Graham and White, 2002). Methane is a valuable fuel and a potential greenhouse gas, and accordingly, the ability to manipulate the organism genetically and cultivate under controlled conditions in a bioreactor (Miller et al., 1988; Mukhopadhyay et al., 1999) would catalyze efforts for exploiting *M. jannaschii* toward commercial production of methane and studying greenhouse gas emission in a high temperature environment. This is significant as biotechnological production of methane has been extensively studied with mesophilic and the thermophilic methanogens and not with hyperthermophilic isolates (Castellano-Hinojosa et al., 2018). In conclusion, the advancement presented in this report strengthens the position of *Methanocaldococcus jannaschii* as an important model for studies on archaea, hyperthermophilic metabolisms, and evolutionary biology and makes it attractive for applied purposes.

## Dedication

Dedicated to the memory of Professor Ralph S. Wolfe (1921–2019).

## Author Contributions

DS and BM designed the research, analyzed the data, and wrote the paper. DS and MF performed the research.

### Conflict of Interest Statement

The authors declare that the research was conducted in the absence of any commercial or financial relationships that could be construed as a potential conflict of interest.
