# Corrigendum: A Genetic System for *Methanocaldococcus jannaschii*: An Evolutionary Deeply Rooted Hyperthermophilic Methanarchaeon

**DOI:** 10.3389/fmicb.2021.649127

**Published:** 2021-03-09

**Authors:** Dwi Susanti, Mary C. Frazier, Biswarup Mukhopadhyay

**Affiliations:** ^1^Department of Biochemistry, Virginia Tech, Blacksburg, VA, United States; ^2^Biocomplexity Institute, Virginia Tech, Blacksburg, VA, United States; ^3^Virginia Tech Carilion School of Medicine, Virginia Tech, Blacksburg, VA, United States

**Keywords:** genetics, ancient archaea, methanogen, hyperthermophile, anaerobe, *Methanocaldococcus jannaschii*, gene knockout, protein overexpression

In the original article, the reference for Balch et al. (1979) was incorrectly written as Balch, W. E., and Wolfe, R. S. (1976). New approach to the cultivation of methanogenic bacteria: 2-mercaptoethanesulfonic acid (HS-CoM)-dependent growth of *Methanobacterium ruminantium* in a pressureized atmosphere. *Appl. Environ. Microbiol*. 32, 781–791. It should be Balch, W. E., Fox, G. E., Magrum, L. J., Woese, C. R., and Wolfe, R. S. (1979). Methanogens: reevaluation of a unique biological group. *Microbiol. Rev*. 43, 260–296.

The 2 L steel anaerobic canister used in our study was a modified version of a pressure cylinder described in Figure 5 of the above-mentioned publication (Balch et al., 1979). The length or the height of the modified unit was 17.5 cm.

The authors apologize for this error and state that this does not change the scientific conclusions of the article in any way. The original article has been updated.
