# Supplementary material for: A Genetic System for Methanocaldococcus jannaschii: An Evolutionary Deeply Rooted Hyperthermophilic Methanarchaeon
Source: Front Microbiol. 2019 Jul 3;10:1256. doi: 10.3389/fmicb.2019.01256 (PMC6616113; doi:10.3389/fmicb.2019.01256)
Supplement: Supplementary file 1 [file Data_Sheet_1.pdf]

Supplementary Materials

A Genetic System for *Methanocaldococcus jannaschii*, an Evolutionary Deeply Rooted Hyperthermophilic Methanarchaeon

Dwi Susanti[1], Mary C. Frazier[1], Biswarup Mukhopadhyay[1, 2, 3*]

[1]Department of Biochemistry, Virginia Tech, Blacksburg, VA, USA

[2]Biocomplexity Institute, Virginia Tech, Blacksburg, VA, USA

[3]Virginia Tech Carilion School of Medicine, Virginia Tech, Blacksburg, VA, USA

[*]**Correspondence:**
Biswarup Mukhopadhyay
biswarup@vt.edu

Keywords: Genetics, ancient archaea, methanogen, hyperthermophile, anaerobe, *Methanocaldococcus jannaschii,* gene knock out, protein overexpression

1 Supplementary Figure and Tables

1.1 Supplementary Figure

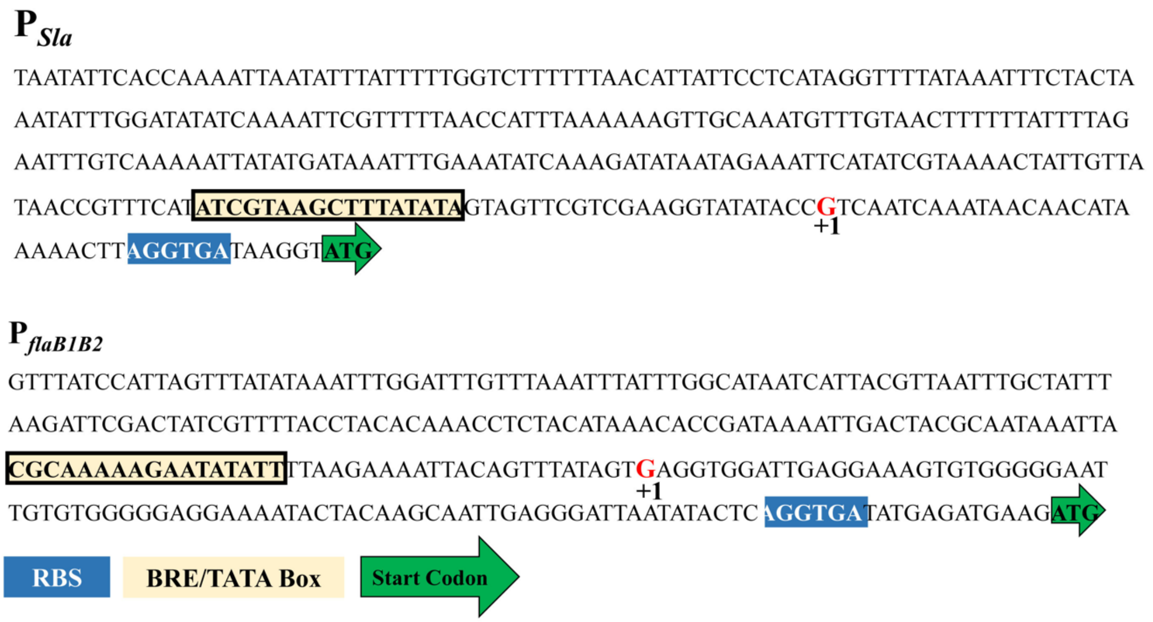

Figure S1. DNA sequences of promoters used in the study. Promoter name, respective gene or operon: P_{sla}, S-layer protein gene of *Methanocaldococcus* sp. FS406-22 (locus tag number, *mfs*40622_1341); $P_{flaB1B2}$, flagellin B1B2 operon of *Methanocaldococcus jannaschii* (locus tag number, *mj*_0891-2). Indicated BRE/TATA box regions and the transcription start sites are either directly based on or inferred from published work with *M. jannaschii* (4, 5). +1, transcription start site.

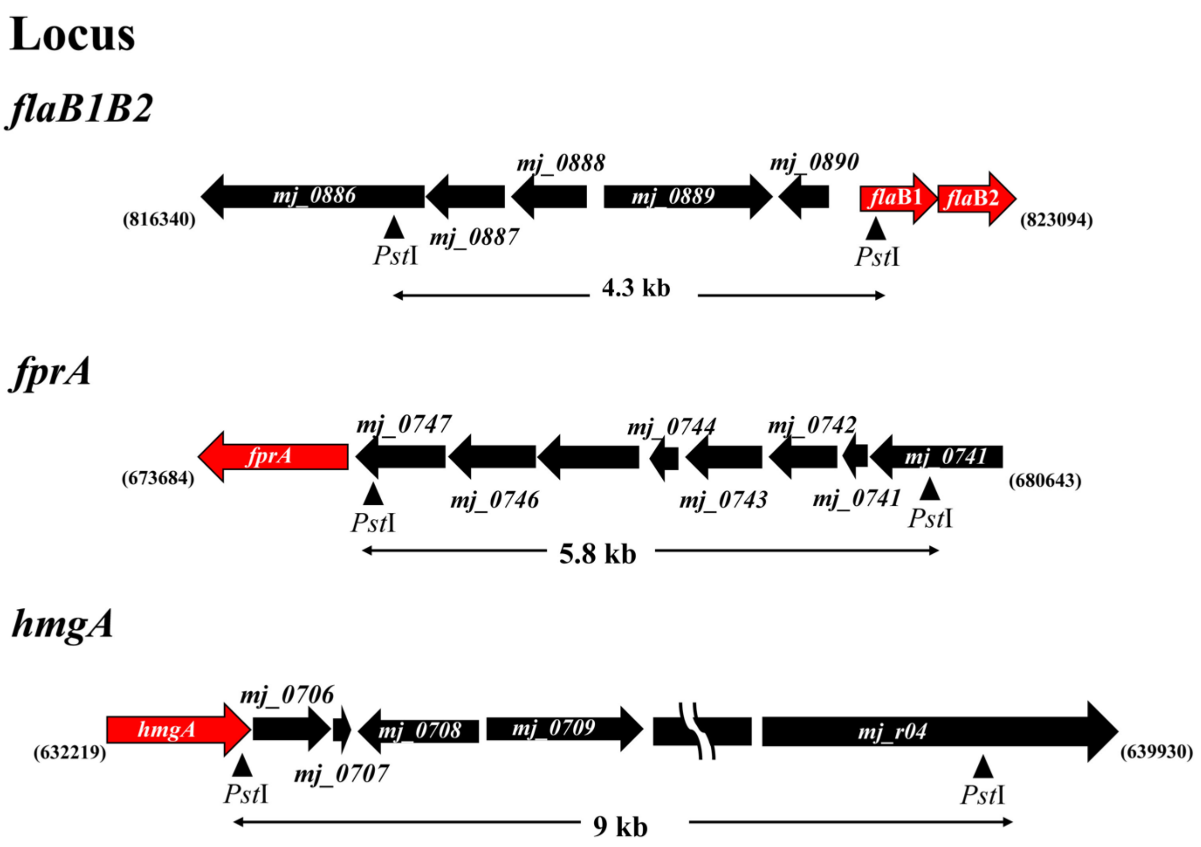

Figure S2. *Pst*I restriction map for the select regions of *M. jannaschii* genome. This information was used to assign identities to the bands a-c in the Southern blot (Fig. 3C). The expected sizes of the fragments resulting from *Pst*I digestion are shown. These fragments lack *Sac*I restriction sites.

```
  1 MDYKDHDGDY KDHDIDYKDD DDKGSAASWS HPQFEKGGGS GGGSGGGSWS
 51 HPQFEKSGMK KYESRRSKIA DGVYWVGVLD WDIRMYHGYT LKGTTYNAYL
101 VFGDEKVALI DNTYPGTSAQ MWGRIKDAFE KEGREFKIDV IVQNHVEKDH
151 SGALPEIHKK FPDAPIYCTE VAVEGLKKHY PSLKDAQFKV VHTGDTVDLG
201 GKTLTFLEAP LLHWPDSMFT FYNEGGILFS NDAFGQHLCF PAHKRFDKDI
251 PEYVLMDANQ KFYANLITPL SKLVLKKFEE VIQLGLLEKI KMIAPSHGQI
301 WTDPMKVIKA YQDFATGKAA KDKAVIVYDT MHYSTQKMAH AFAEGLMSEG
351 IDVVMYFLHY DERSEIVKDI LDAKAVLFGI PTIYDEPYPS IGDIIYYLRG
401 LKFNRTGFKR LAVTFGSMGG EGGAVAKIAE DLAKCGFEVI NQYELYYVPT
451 EDELTNCYNM GKELAKRIKE MKIE
```

Figure S3. Regions of FprA represented by the thermolysine peptides identified in MALDI mass spectrometric analysis. Red, areas covered in the analysis, respectively; underlined, 3xFLAG tag; underlined and italics, strep-tag. The sequence coverage in the analysis, 261/474 or 55%.

1.2 Supplementary Tables

Table S1. Primers and synthetic DNA

Name	DNA sequence (5’ -> 3’)
Primer 1	CTCAAAGGAATAATCCTCTG
Primer 2	GGAACATTCAGACCAGAAAG
Primer 3	GTGGTTTTAGCATACCTATCTGG
Primer 4	GCATAAAACTTCTGGTTTGC
3xFLAG-2xSTREP	ATGGACTATAAGGATCACGATGGAGATTATAAAGATCATGATATTGATTATAAGGATGACGATGATAAAGGATCAGCTGCTTCATGGTCACACCCACAATTTGAAAAAGGAGGTGGATCAGGAGGTGGTTCTGGTGGAGGATCTTGGTCTCATCCACAATTCGAGAAGTCAGGA

Table S2. DNA sequences of coding regions, promoters, and a terminator

Sequence identity<br>(locus tag number)[a]	DNA sequence (5’ -> 3’)
hmgA<br>*(mj_0705)*	ATGGAAAATTATAATGACATCCTTGAAAAAATGTTAAATGGAGAAATAAAGCCATATCAATTAG<br>ATAAAATGTTTGGTTCAAAAATAGCAACTGAAATTAGAAGAAAATTTATTGAAAAAAAGGTTGG<br>GATTGAATTTAAGCATATATGCAATTACTCAATAGATGAAGAAATGGCTATGAAGAAGAATATA<br>GAAAATATGATTGGAGCTATACAGATTCCATTGGGTTTTGCTGGGCCTTTAAAGATAAATGGGG<br>AATATGCAAAGGGAGAGTTTTACATCCCATTGGCAACAACTGAAGGAGCTTTGGTGGCATCGGT<br>TAATAGGGGTTGCTCAATAATAACAAAATGTGGGGGGGCAACTGTTAGGGTTATAGATGATAAG<br>ATGACAAGAGCCCCTTGCTTAAAAACAAAGAGTGTTGTAGATGCAATAAAAGTCAGAGATTGGA<br>TTAGAGAAAACTTTGAGAGGATAAAGGAAGTTGCTGAATCAACAACAAGGCATGGAAAGCTAAT<br>AAAGATAGAGCCAATTTTAATCGTTGGAAGAAATCTATATCCAAGATTTGTATTTAAAACTGGA<br>GATGCCATGGGCATGAATATGGTTACAATTGCAACAGAGAAGGCATGTAATTTTATAGAGGGGG<br>AATTAAAAAAAGAAGGCATATTTGTTAAAACAGTTGCTGTCAGTGGAAATGCATGTGTAGATAA<br>AAAGCCTAGTGGAATGAACTTAATTAATGGTAGAGGGAAATCTATTGTAGCAGAGGTATTTTTA<br>ACTGAGAAGGAAGTTAATAAATACCTAAAAACCACTTCCCAAGCTATAGCTGAAGTAAATAGGT<br>TAAAGAATTATATAGGTTCAGCAATAAGCAATTCAATGGGATTCAATGCCCATTATGCAAATAT<br>CATTGGGGCAATATTCTTAGCTACTGGGCAAGATGAGGCACATATAGTTGAGGGTAGTTTAGGA<br>ATTACAATGGCTGAAGTTGAAGATGATGGATTATATTTTTCAGTAACTCTCCCAGATGTTCCTA<br>TTGGGACTGTTGGAGGAGGAACAAGGGTTGAAACACAAAAAGAGTGCTTAGAGATGCTTGGTTG<br>CTACGGAGATAATAAAGCTTTAAAATTTGCTGAAATTGTTGGAGCTGCAGTTTTAGCTGGGGAG<br>TTATCTTTATTAGGAGCTTTAGCAGCTGGACATTTAGGAAAAGCTCATCAAGAACTCGGTAGAT<br>AA
fprA<br>*(mj_0748)*	ATGAAAAAATATGAAAGCAGACGCAGCAAAATAGCTGATGGTGTATATTGGGTGGGGGTTTTAG<br>ACTGGGACATAAGAATGTATCACGGCTACACATTAAAAGGAACAACATACAATGCCTATTTAGT<br>CTTTGGAGATGAAAAAGTTGCTTTAATAGACAACACATACCCAGGAACCTCCGCTCAAATGTGG<br>GGGAGGATAAAAGATGCATTTGAAAAAGAGGGGAGGGAATTTAAAATTGATGTAATCGTTCAAA<br>ACCACGTAGAAAAAGACCACAGTGGAGCTCTCCCTGAAATACACAAAAAATTCCCAGATGCACC<br>AATATACTGTACTGAGGTAGCTGTTGAGGGACTTAAAAAGCACTATCCATCATTAAAAGACGCT<br>CAATTTAAGGTTGTTCATACAGGAGATACAGTTGATTTAGGAGGAAAGACATTAACATTCTTAG<br>AAGCTCCTCTATTACACTGGCCAGATAGTATGTTTACCTTCTACAACGAAGGGGGAATTTTATT<br>CTCAAACGATGCATTTGGACAGCATCTCTGCTTCCCAGCACACAAGAGATTTGATAAAGATATT<br>CCAGAGTATGTGTTAATGGATGCAAACCAGAAGTTTTATGCTAATTTAATTACTCCACTGTCAA<br>AGCTTGTATTAAAGAAATTTGAGGAAGTTATTCAGTTGGGATTATTAGAAAAGATAAAAATGAT<br>TGCCCCATCACACGGGCAGATATGGACAGACCCAATGAAAGTTATTAAGGCATATCAAGACTTT<br>GCTACTGGTAAAGCAGCTAAGGATAAGGCAGTTATCGTTTATGATACTATGCACTACTCAACAC<br>AAAAGATGGCTCATGCATTTGCAGAGGGTTTAATGAGTGAGGGAATTGATGTTGTAATGTATTT<br>CTTACACTACGATGAGAGAAGTGAGATTGTTAAAGACATCTTAGATGCTAAGGCAGTTCTCTTT<br>GGAATTCCAACAATCTATGATGAGCCATATCCATCAATTGGAGATATCATATACTACTTGAGAG<br>GATTGAAATTTAACAGAACAGGATTTAAGAGATTGGCGGTTACTTTTGGTTCAATGGGGGGAGA<br>AGGTGGAGCAGTTGCTAAGATTGCTGAAGACTTGGCGAAATGTGGATTTGAAGTTATTAATCAA<br>TATGAACTCTACTATGTCCCAACAGAGGATGAATTAACAAACTGCTACAATATGGGTAAAGAAT<br>TGGCTAAGAGAATTAAAGAGATGAAGATTGAATGA
P_{sla}<br>(*MFS40622_1341* for *sla*)	AAATATTCACCAAAATTAATATTTATTTTTGGTCTTTTTTAACATTATTCCTCATAGGTTTTAT<br>AAATTTCTACTAAATATTTGGATATATCAAAATTCGTTTTTAACCATTTAAAAAGTTGCAAAT<br>GTTTGTAACTTTTTTATTTTAGAATTTGTCAAAAATTATATGATAAATTTGAAATATCAAAGAT<br>ATAATAGAAATTCATATCGTAAAACTATTGTTATAACCGTTTCATATCGTAAGCTTTATATAGT<br>AGTTCGTCGAAGGTATATACCGTCAATCAAATAACAACATAA
P_{flaB1}<br>(*mj_0891* for *fla*B1)	GTTTATCCATTAGTTTATATAAATTTGGATTTGTTTAAATTTATTTGGCATAATCATTACGTTA<br>ATTTGCTATTTAAGATTCGACTATCGTTTTACCTACACAAACCTCTACATAAACACCGATAAAA<br>TTGACTACGCAATAAATTACGCAAAAAGAATATATTTTAAGAAAATTACAGTTTATAGTGAGGT<br>GGATTGAGGAAAGTGTGGGGGAATTGTGTGGGGGAGGAAAATACTACAAGCAATTGAGGGATTA<br>ATATACTCAGGTGATATGAGATGAAG
T_{sla}<br>(*MFS40622_1341* for *sla*)	ATCCTCTAAATCTTTTTTTATTTTTTTAGATGAC

[a] P, promoter; T, terminator; *hmg*A, *fpr*A, *sla*, and *fla*B1, genes for HMG CoA reductase, $F_{420}H_2$ oxidase, S-layer protein, and flagellin, respectively.

REFERENCES

1. **Johnson EF, Mukhopadhyay B.** 2005. A new type of sulfite reductase, a novel coenzyme F_{420}-dependent enzyme, from the methanarchaeon *Methanocaldococcus jannaschii*. J Biol Chem **280:**38776-38786.
2. **Mukhopadhyay B, Johnson EF, Wolfe RS.** 1999. Reactor-scale cultivation of the hyperthermophilic methanarchaeon *Methanococcus jannaschii* to high cell densities. Appl Environ Microbiol **65:**5059-5065.
3. **Balch WE, Wolfe RS.** 1976. New approach to the cultivation of methanogenic bacteria: 2-mercaptoethanesulfonic acid (HS-CoM)-dependent growth of *Methanobacterium ruminantium* in a pressureized atmosphere. Appl Environ Microbiol **32:**781-791.
4. **Zhang J, Li E, Olsen GJ.** 2009. Protein-coding gene promoters in *Methanocaldococcus* (*Methanococcus*) *jannaschii*. Nucleic Acids Res **37:**3588-3601.
5. **Smollett K, Blombach F, Reichelt R, Thomm M, Werner F.** 2017. A global analysis of transcription reveals two modes of Spt4/5 recruitment to archaeal RNA polymerase. Nat Microbiol **2:**17021.
